# Draft Genome Sequence of Multidrug-Resistant Enterococcus faecium Strain E1298, with a Sequence Type 1274 Profile, Recovered from the Cloacal Microbiome of a Tropical Screech Owl (Megascops choliba) in Rio de Janeiro, Brazil

**DOI:** 10.1128/MRA.00168-19

**Published:** 2019-04-18

**Authors:** Andréa Andrade Rangel Freitas, Stephanie Silva Rodrigues Souza, Adriana Rocha Faria, Paul J. Planet, Vânia Lúcia Carreira Merquior, Lúcia Martins Teixeira

**Affiliations:** a Instituto de Microbiologia Paulo de Góes, Universidade Federal do Rio de Janeiro, Rio de Janeiro, RJ, Brazil; b Faculdade de Ciências Médicas, Universidade do Estado do Rio de Janeiro, Rio de Janeiro, RJ, Brazil; c Faculdade de Medicina, Universidade Federal do Rio de Janeiro, Rio de Janeiro, RJ, Brazil; d Perelman School of Medicine, University of Pennsylvania, Philadelphia, Pennsylvania, USA; e Pediatric Infectious Disease Division, Children’s Hospital of Philadelphia, Philadelphia, Pennsylvania, USA; f Sackler Institute for Comparative Genomics, American Museum of Natural History, New York, New York, USA; University of Maryland School of Medicine

## Abstract

Here, we present the draft genome sequence of Enterococcus faecium strain E1298, a representative of the clonal complex 17 (CC17), identified as sequence type 1274 (ST1274) and resistant to multiple classes of antimicrobials, isolated from the cloaca of a tropical screech owl (Megascops choliba) in Rio de Janeiro, Brazil.

## ANNOUNCEMENT

Certain multidrug-resistant lineages of Enterococcus faecium are recognized as important agents of health care-associated infections and public health threats (1–3). Most of these lineages belong to clonal complex 17 (CC17), which is disseminated throughout the world (3–5). Carriage of high-risk E. faecium strains by wild birds has been recently reported (6–8), but the role of birds in the dissemination of these microorganisms is still uncertain.

Here, we present the draft genome sequence of Enterococcus faecium E1298, a multidrug-resistant strain belonging to sequence type 1274 (ST1274), a member of CC17 (http://pubmlst.org/efaecium). The strain was isolated from a tropical screech owl (*Megascops choliba*), a common owl in urban environments, that was admitted in October 2013 to a wildlife rehabilitation center in Rio de Janeiro, Brazil. A cloacal content sample was collected using a swab (Transystem with Amies medium; Copan, Brescia, Italy) and inoculated into Enterococcosel broth (BD Microbiology Systems, Cockeysville, MD, USA). After incubation for 24 h at 37°C, an aliquot of the broth culture was streaked onto an Enterococcosel agar plate (BD Microbiology Systems) and incubated under the same conditions.

For DNA extraction, a single bacterial colony grown on a blood agar plate was inoculated into 5 ml of tryptic soy broth and incubated overnight at 37°C. Genomic DNA was obtained from 1.5 ml of that culture using a Wizard genomic DNA purification kit (Promega, Madison, WI, USA), prepped using the Nextera XT kit, and sequenced on a HiSeq 2500 sequencer (Illumina, Inc., San Diego, CA, USA) with 125-bp paired-end reads. A total of 344,111 paired-end Illumina reads and 51,960,761 bp were obtained. Reads were trimmed using Trimmomatic 0.38 (9), and quality metrics were assessed by FastQC v0.11.18 (10). High-quality reads were assembled by the autoassembly strategy and annotated using the RAST tool kit (RASTtk) in the genome annotation service of the Pathosystems Resource Integration Center (PATRIC 3.4.11) (11), leading to the prediction of 51 tRNAs and 3 rRNAs and the identification of 2,945 coding sequences (CDS). The draft genome has a total size of 2,897,331 bp with a G+C content of 37.79% and consists of 117 contigs (*N*_50_ length of 59,074 bp), with a coverage of 18×.

A comparison of amino acid polymorphisms detected in the C-terminal region of PBP5 (coded by the *pbp5* gene) with a reference sequence (GenBank accession number X84860) showed the polymorphisms Met485Ala, Ala499Thr, Glu629Val, and Pro667Ser and the insertion of serine after position 466, associated, when combined, with high-level resistance to β-lactam antibiotics in E. faecium strains from animals and humans (1, 12). Alignment of the *parC* and *gyrA* genes with the E. faecium DO genome (GenBank accession number CP003583) and quinolone resistance-determining regions (QRDRs) (GenBank accession numbers AF060881 and AB017811) revealed single amino acid polymorphisms in codons 82 (Ser to Ile) and 84 (Ser to Arg), respectively.

Using the ResFinder 3.1 tool (13), we identified the following genes: *ant(6)-Ia*, *aph(2’)-Id*, *aph(3’)-III*, and *sat-4* (aminoglycoside resistance); *msrC* and *erm(B)* (macrolide, lincosamide, and streptogramin B resistance); *lnu*(B) (lincosamide resistance); and *tet*(M) (tetracycline resistance). The *acm* and *efaAfm* virulence genes were identified by VirulenceFinder 2.0 (14). Plasmids of the rep1, repUS15, and rep14 families were detected using PlasmidFinder 2.0 (15), and Phast (16) predicted two incomplete prophage regions. No sequences associated with a CRISPR region were identified by using the CRISPRFinder (17).

### Data availability.

This whole-genome shotgun project has been deposited at DDBJ/ENA/GenBank under the accession number RCFR00000000. The version described in this paper is the first version, RCFR01000000. Raw sequence reads have been deposited in the NCBI Sequence Read Archive (SRA) under the BioProject accession number PRJNA494878.
